# Microbiological mechanisms of immune response modulation: clinical implications

**DOI:** 10.3389/fimmu.2026.1927480

**Published:** 2026-10-02

**Authors:** Aleksander Deptuła, Aleksandra Ćwirko-Godycka, Agata Majcherczak, Amanda Zoń, Wiktoria Rosiek, Katarzyna Napiórkowska-Baran

**Affiliations:** 1 Department of Propaedeutics of Medicine and Infection Prevention, Collegium Medicum, Bydgoszcz, Poland; 2 Tczew Hospitals S.A., Tczew, Poland; 3 University Hospital No. 2 Dr Jan Biziel, Bydgoszcz, Poland; 4 Student Research Club of Clinical Immunology, Department of Allergology, Clinical Immunology and Internal Diseases, Collegium Medicum Bydgoszcz, Nicolaus Copernicus University, Bydgoszcz, Poland; 5 Department of Allergology, Clinical Immunology and Internal Diseases, Collegium Medicum in Bydgoszcz, Nicolaus Copernicus University, Toruń, Poland

**Keywords:** dysbiosis, gut-organ axes, immune modulation, immunotherapy, microbiome, microbiota, mucosal immunity, short-chain fatty acids

## Abstract

The human microbiota is a major regulator of immune development, mucosal barrier integrity, and systemic immune tone. Its effects are mediated by microbe-associated molecular patterns, pattern-recognition receptors, microbial metabolites such as short-chain fatty acids, tryptophan-derived indoles, secondary bile acids and vitamin-pathway metabolites, and direct interactions with epithelial and immune cells. A balanced microbiota supports regulatory T-cell responses, secretory IgA production, innate-like lymphocyte maturation and epithelial barrier function, whereas dysbiosis may promote barrier dysfunction, inflammatory polarization and abnormal responses to dietary, environmental or self-antigens. This narrative review integrates mechanistic and translational evidence across early-life immune maturation, innate and adaptive immune sensing, microbial metabolites, cross-kingdom interactions, gut-organ axes, immune-mediated diseases and microbiota-targeted interventions. Particular emphasis is placed on distinguishing mechanistic evidence from association. Although microbiome-directed therapies are promising, routine clinical translation requires standardized sampling and multi-omics workflows, controlled interventional studies, mechanistic validation, reproducible biomarkers and indication-specific evidence.

## 1 Introduction

The microbiota is no longer viewed as a passive microbial community residing on mucosal surfaces. It is a dynamic ecological and functional system that continuously interacts with epithelial, metabolic, neuroendocrine and immune pathways. The gastrointestinal tract contains the largest microbial ecosystem, but clinically relevant microbial communities also exist on the skin, in the respiratory tract, oral cavity and urogenital tract. These communities shape immune function through microbial antigens, structural molecules and metabolites that calibrate host defence, tissue repair and immune tolerance (1, 2, 56).

At intestinal homeostasis, several cellular compartments operate as an integrated mucosal network. Intestinal epithelial cells, goblet cells and Paneth cells provide physical and chemical barrier functions; dendritic cells and resident macrophages sample microbial signals while limiting unnecessary inflammation; group 3 innate lymphoid cells (ILC3s) support IL-22-dependent epithelial defence; regulatory T cells constrain effector responses; and IgA-producing plasma cells promote antigen exclusion and microbial compartmentalization. Innate-like lymphocytes, including MAIT and natural killer T cells, provide an additional interface between microbial metabolites and rapid immune responses. Microbial communities enriched in *Bifidobacterium* during infancy and selected *Bacteroides* and anaerobic Firmicutes later in life contribute metabolites and structural signals that support these homeostatic processes (10, 21, 23, 31, 33, 56, 57).

In physiological conditions, microbial stimulation supports a state of controlled immune readiness. This state is sufficient to maintain barrier defence and rapid pathogen recognition but restrained enough to avoid excessive inflammation against commensal microorganisms and dietary antigens. When this equilibrium is disrupted, dysbiosis may contribute to chronic inflammation, allergic sensitization, autoimmunity, impaired infection control and altered antitumor immunity. The direction of this relationship is often complex: microbiota changes may contribute to disease, but disease activity, medication, diet and inflammation can also reshape the microbiota (1, 2, 67, 68).

The concept of the holobiont remains useful as a broad biological framework because it emphasizes the integration of host physiology and microbial ecology. However, in a clinically oriented immunology review, this concept should not replace mechanistic precision. The key question is how microbial signals influence epithelial integrity, antigen presentation, cytokine networks, immune-cell differentiation and disease phenotypes (3, 4).

Several recent reviews have addressed microbiota-immunity interactions, microbial metabolites or microbiota-targeted treatment strategies separately (1, 2, 100, 105). The rationale for the present review is to integrate these domains within a single immunological framework that links early-life immune education, innate and adaptive sensing, microbial metabolites, barrier biology, cross-kingdom interactions, gut-organ axes and translational interventions. We additionally distinguish mechanistic or interventional evidence from descriptive associations throughout the review, because failure to separate correlation from causation remains a major limitation of microbiome research.

## 2 Narrative review methodology

### 2.1 Literature search framework

This article was designed as a narrative review rather than a systematic review. A structured but non-exhaustive literature search was performed in PubMed/MEDLINE, Scopus and Web of Science for publications available up to June 2026. Search terms were iteratively combined according to the thematic scope of each section and included microbiota, microbiome, immune modulation, immune response, dysbiosis, gut microbiota, short-chain fatty acids, SCFA, tryptophan metabolism, aryl hydrocarbon receptor, intestinal barrier, secretory IgA, trained immunity, MAIT cells, gut-lung axis, gut-brain axis, gut-skin axis, inflammatory bowel disease, allergy, autoimmunity, inborn errors of immunity and cancer immunotherapy. Boolean operators (AND, OR) were used as appropriate, and additional publications were identified by manual screening of reference lists.

### 2.2 Source selection and scope

Original research articles, systematic reviews, meta-analyses, consensus statements and high-quality narrative reviews published in peer-reviewed journals were considered. Priority was given to studies providing mechanistic evidence, longitudinal observations, controlled interventions, translational relevance or clinically meaningful immune outcomes. Conference abstracts, editorials, non-peer-reviewed publications, studies focused solely on taxonomy without immunological interpretation, and reports with insufficient methodological information were not used as core evidence. The final narrative synthesis included 110 publications. Because the review was not designed as an exhaustive systematic review, no protocol registration, PRISMA flow diagram or formal systematic risk-of-bias assessment was performed.

### 2.3 Narrative synthesis and evidence appraisal

Evidence was organized into predefined mechanistic themes: early-life immune maturation; innate immune recognition and trained responses; adaptive immunity; microbial metabolites; epithelial barrier integrity and microbial translocation; bacterial, viral and fungal communities; dysbiosis in immune-mediated diseases; gut-organ communication; and translational interventions. Study quality was appraised qualitatively according to design, sample size, reproducibility, mechanistic depth and consistency with other evidence. Greater weight was assigned to controlled interventions, longitudinal cohorts, systematic syntheses and mechanistic experimental studies. When findings were inconsistent, the direction and strength of evidence were explicitly qualified rather than pooled quantitatively.

## 3 Early-life microbiota and immune maturation

The first years of life represent a critical window for immune education. Microbial colonization begins in the context of maternal microbial exposure and becomes rapidly shaped by delivery mode, gestational age, breastfeeding, antibiotic exposure, diet, siblings, pets, geography and environmental microbial diversity. These factors influence not only which microorganisms are present but also their metabolic output and the timing of exposure to the developing mucosal immune system (5–9). The process of microflora formation begins with the spread of microorganisms during childbirth and after birth, the newborn remains in constant exposure to live bacteria or spores from the mother and other family members (7).

During early colonization, facultative anaerobes initially consume oxygen and facilitate the subsequent expansion of obligate anaerobes. Child’s diet is the most important factor influencing the microbiome. Breastfeeding and human milk oligosaccharides favor *Bifidobacterium*-rich communities, which generate acetate and lactate and can help create an ecological environment that supports epithelial integrity and colonization resistance. *Bacteroides* and other anaerobic taxa expand with dietary diversification, increasing the range of microbial metabolites available to the host. Caesarean delivery, prematurity and perinatal antibiotics may delay or alter these trajectories, although ecological recovery is possible and varies between individuals (5–9, 57).

Immune maturation is coordinated across epithelial and hematopoietic compartments. Commensal colonization promotes regulatory T-cell responses and helps establish mutualism, while dendritic cells and macrophages integrate microbial signals with epithelial-derived mediators. Secretory IgA restricts microbial adhesion and antigen access without requiring destructive inflammation. ILC3-derived IL-22 supports epithelial repair, antimicrobial peptide production and containment of luminal microorganisms. These processes collectively shift the neonatal immune system from a predominantly tolerance-oriented state toward a more mature balance between tolerance and antimicrobial responsiveness (10–12, 31–33).

Early microbial exposure also shapes innate-like lymphocyte compartments. Olszak et al. showed that microbial exposure during a defined early-life window had persistent effects on natural killer T-cell accumulation and inflammatory susceptibility (11). In murine models, commensal riboflavin biosynthesis is also required for normal intrathymic MAIT-cell development: the microbial metabolite 5-OP-RU can reach the thymus, bind MR1 and promote expansion and maturation of MAIT-cell precursors.

These observations provide a mechanistic link between microbial metabolism during early life and the development of non-conventional T-cell populations (107).

The interaction between microbiota and mucosal IgA is particularly relevant to allergic disease. Higher intestinal IgA has been associated with a lower risk of IgE-associated allergic outcomes, whereas altered IgA recognition of the gut microbiota can precede childhood allergy and asthma (34, 35). More recent data comparing infants from traditional farming and urban environments similarly suggest that patterns of microbial IgA coating and maternal milk IgA responses differ between populations with markedly different allergic outcomes. These studies do not establish a single protective microbiota, but they support the concept that the quality of early mucosal recognition may be as important as bacterial abundance alone (106).

The clinical relevance of early microbial exposure is therefore better understood through a microbiota-centered extension of the hygiene or ‘old friends’ hypothesis. The concept does not imply that reduced hygiene is protective; rather, it proposes that loss of appropriate microbial diversity and timing of non-pathogenic exposure may impair the maturation of regulatory networks in genetically susceptible individuals (13–15, **Figure 1**). These major determinants of early-life microbiota and their immunological consequences are summarized in **Table 1**.

**Figure 1:**
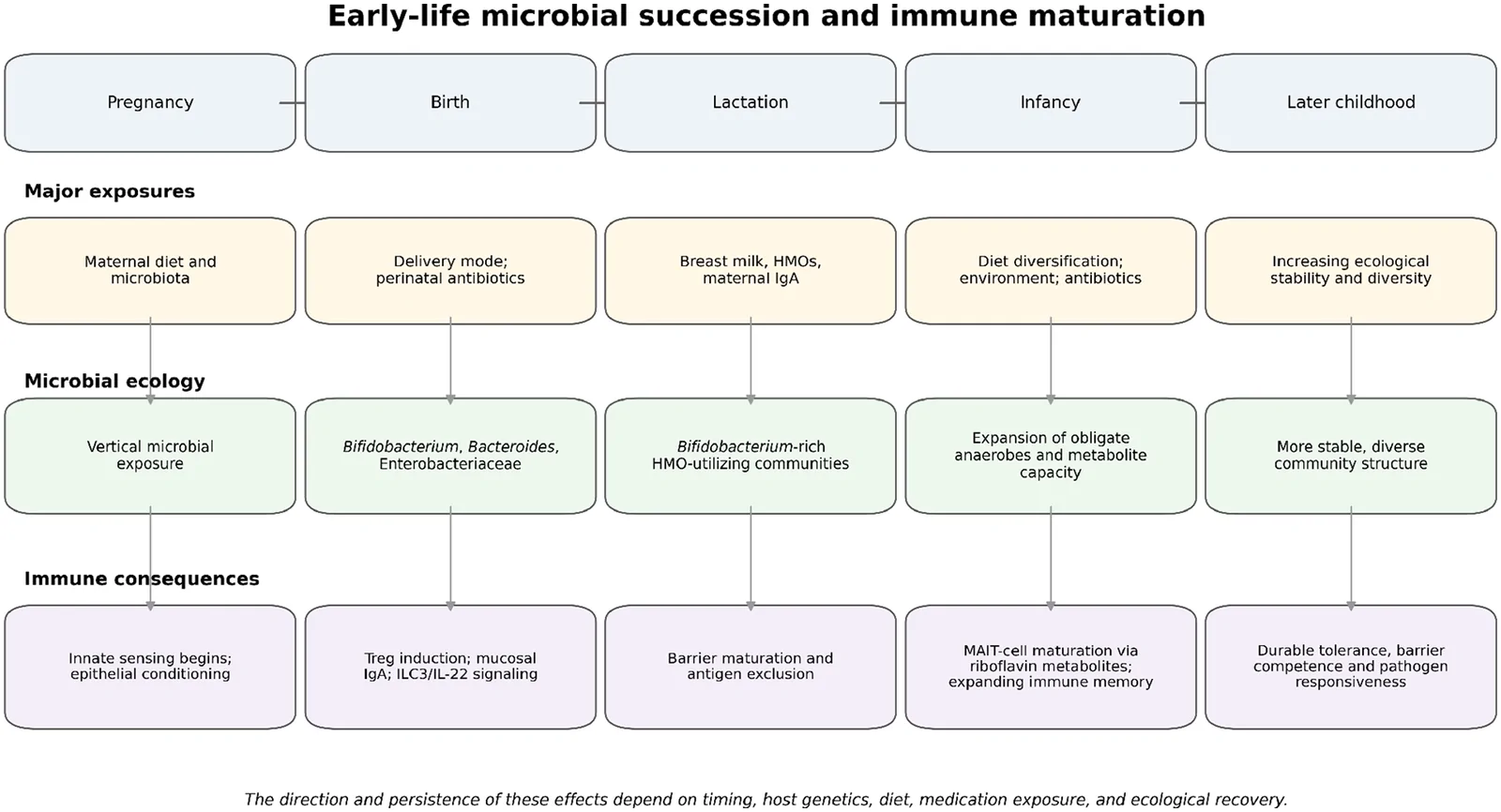
Original schematic summarizing early–life exposures, microbial succession and immune maturation. The image was created *de novo* for the revised manuscript on the basis of the literature discussed in Section 3 (5–15, 34, 35, 57, 106, 107).

**Table 1:** Representative early–life microbiota–immune findings.

Exposure/stage	Microbiota feature	Immune consequence	Representative evidence
Breastfeeding/HMOs	*Bifidobacterium*–rich communities	Acetate/lactate production, epithelial and mucosal immune support	(5, 6, 8, 57)
Commensal colonization	Progressive anaerobic succession	Treg induction and mutualistic immune regulation	(10, 12)
Early microbial exposure	Timing–sensitive microbial antigen exposure	Persistent effects on innate–like lymphocyte function	(11)
Riboflavin–producing commensals	5–OP–RU generation	MR1–dependent MAIT–cell development in experimental models	(107)
Mucosal IgA development	Taxon–specific IgA coating patterns	Microbial containment, altered patterns may precede allergy	(34, 35, 106)
Caesarean delivery/perinatal antibiotics	Altered or delayed microbial succession	Potentially altered immune education, effects depend on recovery	(5–9)

## 4 Innate immune recognition and trained responses

Microbial signals are detected by pattern-recognition receptors (PRRs), including Toll-like receptors, NOD-like receptors, RIG-I-like receptors and C-type lectin receptors. These receptors recognize conserved microbial structures, commonly described as microbe-associated molecular patterns (MAMPs), including lipopolysaccharide, lipoteichoic acid, peptidoglycan, flagellin, microbial nucleic acids, beta-glucans and mannans. Their activation engages MyD88-, TRIF-, NF-κB-, IRF- and inflammasome-dependent pathways and thereby regulates cytokine production, type I interferon responses, antimicrobial programs and antigen-presenting cell activation (16–18).

The immunological outcome of PRR signaling depends on anatomical compartmentalization and cellular context. Intestinal epithelial cells integrate PRR signals with nutrient and metabolite sensing while maintaining barrier function. Resident intestinal macrophages are continuously replenished from circulating monocytes but acquire a more regulatory phenotype in the healthy mucosa, with efficient phagocytosis and limited inflammatory cytokine release. Dendritic cells transport sampled antigen to lymphoid tissues and can support either tolerance or effector differentiation depending on the microbial, epithelial and cytokine context (20, 21, 56).

Microbiota-derived signals can also influence trained innate immunity. Unlike antigen-specific adaptive memory, trained immunity reflects durable metabolic and epigenetic reprogramming of innate immune cells or hematopoietic progenitors, which can alter subsequent responses to unrelated challenges. Microbial ligands can modify chromatin accessibility, glycolytic and mitochondrial programs and myeloid-cell responsiveness. This may improve antimicrobial readiness after some exposures but may also amplify chronic inflammatory responses when microbial products persist because of dysbiosis or barrier injury (19, 20).

Microbial metabolites provide an additional layer of control over innate activation and differentiation. Butyrate and propionate can alter macrophage and dendritic-cell cytokine programs through GPCR signaling and HDAC inhibition, whereas tryptophan-derived AhR ligands influence epithelial cells, ILC3s and antigen-presenting cells. Consequently, the same PRR signal can produce different outcomes depending on metabolite availability and the surrounding cytokine milieu (22, 23, 26, 39).

These mechanisms are clinically relevant because loss of compartmentalized microbial sensing is repeatedly observed in inflammatory disease. Expansion of pathobionts or increased permeability may increase exposure to MAMPs, promoting NF-κB activation, inflammasome signaling, inflammatory monocyte recruitment and neutrophil responses in disorders such as IBD and cirrhosis. Conversely, restoration of barrier integrity or microbial metabolic function can reduce this inflammatory input in experimental models. The available clinical evidence, however, does not support treating trained immunity or PRR activation as uniformly beneficial or harmful (53–55, 60, 74, 75).

## 5 Adaptive immunity: T cells, B cells and IgA

The microbiota strongly influences adaptive immunity. CD4+ T-cell differentiation into Th1, Th2, Th17 and regulatory T-cell subsets is shaped by microbial antigens, metabolites, epithelial signals and antigen-presenting cells. Short-chain fatty acids can promote colonic Treg differentiation through histone deacetylase inhibition and epigenetic regulation, but they may also support effector responses under particular cytokine conditions. This duality is clinically important because microbial metabolites cannot be classified as uniformly anti-inflammatory or pro-inflammatory (23–25).

Tryptophan metabolites and secondary bile acids also modulate T-cell responses. Indole derivatives activate AhR-dependent pathways that support IL-22 production, epithelial protection and context-dependent regulatory responses. Secondary bile-acid derivatives can alter the balance between Th17 and Treg differentiation, providing a direct link between bacterial enzymatic activity and adaptive immune polarization (26–28).

CD8+ T cells are influenced by microbial metabolism as well. Acetate and butyrate can support the metabolic fitness and memory potential of antigen-activated CD8+ T cells. In oncology, several gut microbial profiles have been associated with immune-checkpoint-inhibitor responses, potentially through altered antigen presentation, dendritic-cell activation and cytotoxic T-cell recruitment. These associations are biologically plausible but remain variable across tumor types and cohorts (29, 30).

B-cell responses are shaped by microbial antigens, metabolites and mucosal cytokines. Secretory IgA limits microbial adhesion, neutralizes toxins and viruses, restricts antigen access to the epithelium and promotes microbial compartmentalization. IgA is generated through T-cell-dependent and T-cell-independent pathways involving TGF-beta, BAFF and APRIL, while dendritic-cell-derived retinoic acid contributes to gut homing and IgA-secreting plasma-cell differentiation (31–33).

IgA is especially relevant to early immune education and food allergy. Reduced or aberrant IgA responses in infancy may be associated with later allergic outcomes, while effective IgA coating can limit the access of dietary and microbial antigens to the mucosal immune system. This places IgA at the intersection of microbial ecology, epithelial barrier function and adaptive tolerance rather than treating it simply as a marker of mucosal exposure (34–36, 106).

## 6 Microbial metabolites as immune modulators

Short-chain fatty acids (SCFAs), particularly acetate, propionate and butyrate, are generated by bacterial fermentation of dietary substrates. These products play a key role in modulating the immune response and their concentrations vary with diet, microbial composition, intestinal transit, antibiotic exposure and host absorption. SCFAs signal through receptors including GPR41, GPR43 and GPR109A and also act through intracellular pathways such as histone deacetylase inhibition. These mechanisms influence epithelial metabolism, gene transcription and immune-cell differentiation (37–39).

Within the intestine, SCFAs support several mutually reinforcing homeostatic processes. Butyrate is an important energy source for colonocytes, promotes mucus and tight-junction integrity, and can favor regulatory T-cell differentiation and less inflammatory macrophage and dendritic-cell phenotypes. Systemically, SCFAs may influence hematopoiesis, respiratory immunity, neuroimmune signaling and antitumor responses. These effects are dose-, tissue- and context-dependent; concentrations used experimentally may not always reproduce physiological exposure (23–25, 29, 39).

Beyond SCFAs, a second major metabolic interface relies on the bacterial processing of dietary amino acids, most prominently tryptophan. Gut bacteria transform dietary tryptophan into indole and related derivatives, while host pathways generate kynurenine metabolites. Several microbial indoles activate the aryl hydrocarbon receptor (AhR), a transcription factor involved in xenobiotic sensing, barrier integrity and immune regulation. AhR signaling can influence Treg/Th17 balance, ILC3 function, IL-22 production and epithelial repair, thereby linking nutrient availability and microbial enzymatic activity to mucosal immunity (26, 40–43).

This AhR-centered pathway illustrates how a microbial metabolite can connect immune regulation with barrier function. Indole-3-aldehyde and related metabolites can stimulate AhR-dependent IL-22 production, antimicrobial peptide expression and epithelial defence. In parallel, indole derivatives can influence tight-junction and desmosomal organization. Reduced generation of these signals has been implicated in experimental models of mucosal inflammation and infection susceptibility, although the clinical importance of individual indole metabolites remains incompletely defined (26, 43).

In addition to the products of carbohydrate and amino acid metabolism derived from food, the gut microbiota systematically modifies endogenous molecules derived from the host; secondary bile acids are an example of this. These are formed through the microbial dehydroxylation of primary bile acids and act through receptors including FXR, TGR5/GPBAR1, PXR and VDR and can influence macrophage activation, inflammasome signaling, cytokine production and T-cell differentiation. Lithocholic-acid-related metabolites can suppress specific Th17 programs or promote regulatory phenotypes, illustrating how microbial metabolism of a host substrate can reshape adaptive immunity (27, 28, 44, 45).

While low-molecular-weight metabolites interact primarily through metabolic and nuclear receptors, structural bacterial polymers are recognized by the immune system in a fundamentally different way. An uncommon class of these molecules consists of dicationic-dianionic polysaccharides (ZPS). ZPS contain both positively and negatively charged groups and can be processed by antigen-presenting cells for MHC class II presentation to CD4+ T cells. Polysaccharide A (PSA) from *Bacteroides fragilis* is the best-characterized example; it engages TLR2-dependent signaling and promotes IL-10-producing regulatory T-cell responses, while plasmacytoid dendritic cells can contribute to its anti-inflammatory effects. These findings demonstrate that bacterial structural molecules can link innate sensing with antigen-specific adaptive regulation rather than simply trigger inflammation (46, 47).

Riboflavin-pathway metabolites provide a further link between microbial metabolism and unconventional T cells. Many bacteria and yeasts synthesize riboflavin, generating intermediates that can form potent MR1 ligands. 5-OP-RU is an agonistic MR1 ligand that activates mucosal-associated invariant T (MAIT) cells and, in experimental models, contributes to their thymic development. By contrast, 6-formyl pterin (6-FP), a folate-derived MR1-binding metabolite, is non-stimulatory for MAIT cells and can alter MR1 ligand occupancy and availability. These pathways broaden the concept of microbial antigen recognition beyond peptides and lipids to small-molecule metabolites (107, 108).

Taken together, microbial metabolite signaling is best viewed as an interconnected network rather than a collection of isolated pathways. SCFAs alter chromatin and GPCR signaling, indoles engage AhR, bile-acid derivatives act through nuclear and membrane receptors, zwitterionic polysaccharides link TLR2 and MHC-II pathways, and riboflavin-derived ligands activate MR1-restricted T cells (**Figure 2**; **Table 2**).

**Figure 2:**
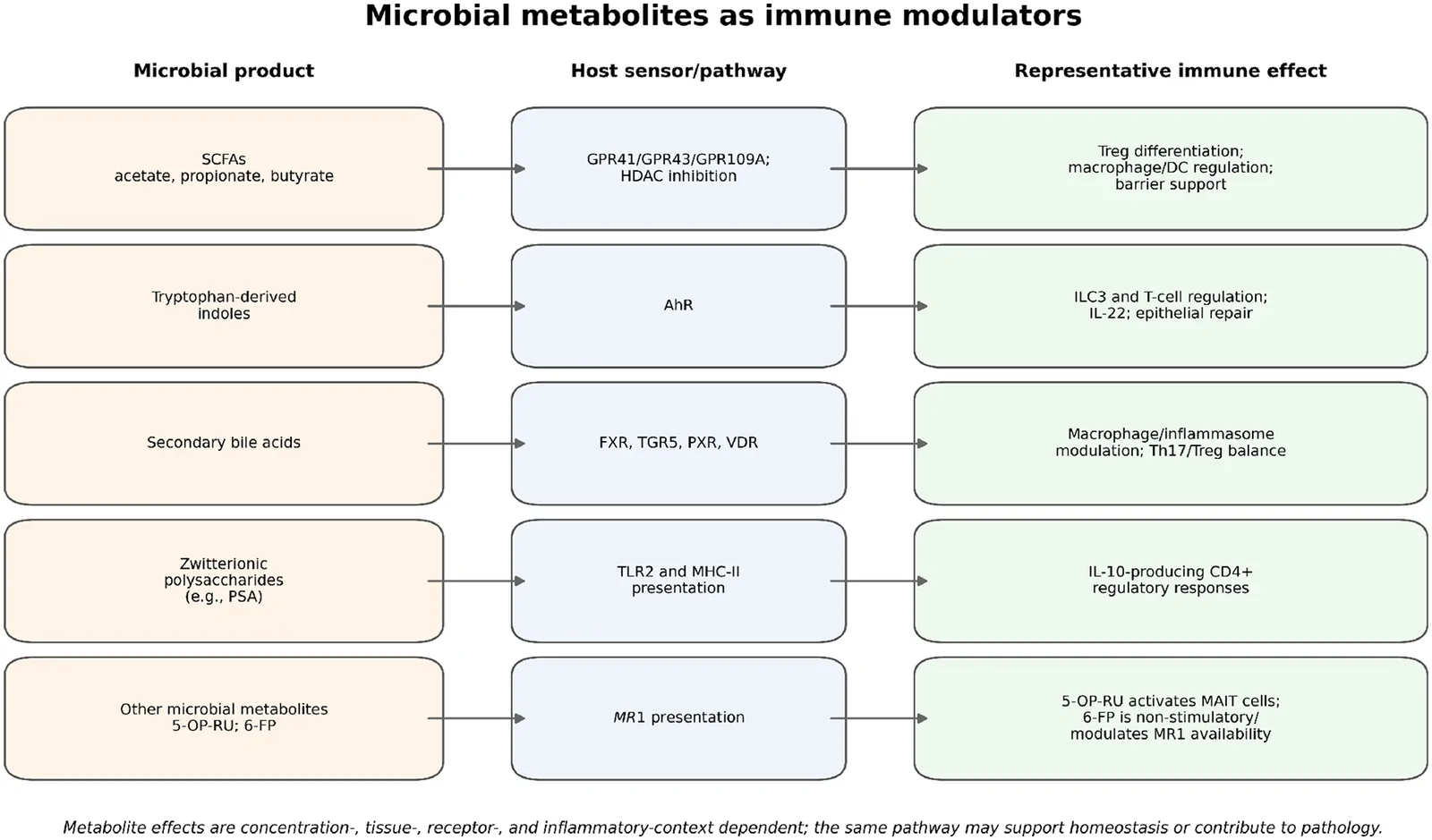
Original schematic of microbial metabolites, host sensing pathways and representative immune effects. The former label “Bacterial Vitamins” has been replaced by “Other microbial metabolites”, 5–OP–RU and 6–FP are described in the text as MR1–binding metabolites with distinct effects on MAIT–cell activation (23–28, 37–47, 107, 108).

**Table 2:** Major microbial metabolites and representative immune pathways.

Metabolite/molecule	Representative microbial source/process	Host sensor/pathway	Principal immune effect	Key references
SCFAs (acetate, propionate, butyrate)	Fermentation of dietary substrates by anaerobic bacteria	GPR41, GPR43, GPR109A, HDAC inhibition	Treg differentiation, macrophage/DC modulation, epithelial support	(23–25, 37–39)
Indole derivatives	Microbial tryptophan metabolism	AhR	ILC3/IL–22 signaling, Treg/Th17 modulation, epithelial repair	(26, 40–43)
Secondary bile acids	Microbial modification of primary bile acids	FXR, TGR5, PXR, VDR	Macrophage and inflammasome modulation, Th17/Treg effects	(27, 28, 44, 45)
Polysaccharide A/zwitterionic polysaccharides	*Bacteroides fragilis* structural polysaccharide	TLR2, MHC class II presentation	IL–10–producing CD4+ regulatory responses	(46, 47)
5–OP–RU	Microbial riboflavin biosynthetic pathway	MR1	MAIT–cell activation and developmental signaling	(107, 108)
6–FP	Folate–derived MR1 ligand	MR1	Non–stimulatory MR1 binding, modulation of ligand occupancy	(108)

## 7 Barrier integrity and bacterial translocation

The intestinal barrier is not merely a permeable wall. It is a multilayered interface composed of mucus, antimicrobial peptides, secretory IgA, epithelial cells, intercellular junctional complexes, lamina-propria immune cells and microbial communities occupying the luminal and mucus-associated niches. This architecture permits controlled immune sampling while restricting uncontrolled penetration of microorganisms and their products (48, 49).

Tight junctions regulate paracellular permeability through proteins including claudins, occludin and zonula occludens proteins. Microbial metabolites, particularly butyrate, can reinforce this organization through several complementary mechanisms. Butyrate provides metabolic fuel to differentiated colonocytes, activates GPCR-dependent signaling, inhibits HDAC activity and can influence HIF-dependent epithelial adaptation. Experimental data also show increased claudin-1 transcription through enhanced SP1 promoter binding and redistribution or increased abundance of ZO-1 and occludin. In inflammatory settings, these effects can oppose cytokine- and LPS-driven disruption of junctional complexes, although the magnitude and direction of the response depend on dose and epithelial context (39, 50–52, 109).

Barrier dysfunction increases exposure of mucosal and systemic immune cells to LPS, peptidoglycan, microbial DNA and other MAMPs. This can activate TLR- and NOD-dependent NF-κB signaling, inflammasomes, inflammatory monocytes and neutrophils. A self-reinforcing cycle may then develop in which inflammation further disrupts junctional integrity and changes the ecological niche, promoting dysbiosis and additional microbial translocation (51–55).

In IBD, depletion of butyrate-producing bacteria and altered SCFA availability may coincide with epithelial metabolic stress, reduced regulatory T-cell support and increased Th17/neutrophil-driven inflammation. In cirrhosis, dysbiosis and impaired barrier integrity facilitate bacterial product translocation into the portal and systemic circulation, contributing to innate immune activation and infectious complications. Metabolic inflammation has similarly been linked to increased systemic exposure to bacterial products, while in autoimmune disorders barrier disruption may amplify antigenic and inflammatory input rather than act as a single initiating cause (53–55, 67, 68, 74, 75).

The absence or reduction of a beneficial metabolite should therefore not be interpreted as a disease-specific deficiency that can simply be replaced therapeutically. For example, lower butyrate availability may remove several homeostatic signals simultaneously—epithelial energy supply, GPCR signaling, HDAC inhibition and Treg support—but clinical responses to direct butyrate supplementation have been heterogeneous. This distinction is important when translating mechanistic observations into therapeutic claims (39, 50, 74).

## 8 Microbial realms: bacteria, pathobionts, viruses and fungi

Commensal bacteria remain the most intensively studied component of the microbiome because they provide a large fraction of the metabolic and antigenic signals discussed above. *Bifidobacterium* species, selected anaerobic *Firmicutes* and *Bacteroides* species can support homeostasis through SCFA production, mucus regulation, IgA stimulation, competitive exclusion and immunoregulatory metabolites. Importantly, these effects are frequently strain- and context-specific, and taxonomic abundance alone does not define function (56, 57).

Pathobionts are resident microorganisms that become harmful under conditions such as barrier injury, immune deficiency, antibiotic exposure or loss of ecological competition. Adherent-invasive *Escherichia coli* in Crohn’s disease provides an example in which bacterial adherence, invasion and survival within macrophages can reinforce inflammatory pathways through sustained NF-κB activation and pro-inflammatory cytokine secretion (IL-1β, TNF-α, IL-23). *Clostridioides difficile* illustrates a different mechanism: antibiotic-mediated loss of colonization resistance allows expansion and toxin-mediated epithelial damage, with subsequent neutrophilic inflammation (58–60).

The virome includes bacteriophages, eukaryotic viruses and endogenous viral elements. Bacteriophages can reshape bacterial abundance, horizontal gene transfer and metabolic capacity, thereby indirectly altering immune exposure to bacterial antigens and metabolites. Phage-mediated lysis of commensal bacteria can lead to a sudden depletion of beneficial metabolites (such as SCFAs) and a simultaneous release of pro-inflammatory bacterial debris (such as LPS and peptidoglycan) and viral nucleic acids can also be detected by RIG-I-like receptors, TLRs (such as TLR3, TLR7, TLR8, and TLR9) and other innate sensors, inducing type I and III interferon responses. In inflammatory disease, changes in the virome may therefore function both as ecological drivers of bacterial communities and as direct immune stimuli (61–63).

The mycobiome is quantitatively smaller than the bacterial microbiome but immunologically potent. Fungal beta-glucans and mannans are recognized by receptors including Dectin-1, the mannose receptor and Toll-like receptors, activating Syk/CARD9-related signaling, phagocytosis, reactive oxygen species, inflammasome pathways and Th17 responses. *Candida albicans* illustrates the importance of morphology: yeast forms can participate in commensalism, whereas hyphal transition increases epithelial interaction and inflammatory signaling. Dysregulated fungal expansion and Dectin-1/CARD9 signaling are not only hallmarks of severe IBD flares, but also significantly contribute to extra-intestinal pathologies, such as spondyloarthritis, primary sclerosing cholangitis (PSC), and systemic autoimmune conditions via molecular mimicry and Th17 cell trafficking (64–66).

Cross-kingdom interactions link these compartments. Antibiotic-induced loss of bacterial SCFA production may facilitate fungal expansion or hyphal growth; bacteriophage-driven changes in bacterial communities can alter metabolite production; and fungal or viral inflammation can modify epithelial permeability and bacterial localization. These processes are relevant to IBD, post-antibiotic states, immunodeficiency and other inflammatory disorders because the immune system responds to the combined antigenic and metabolic output of the ecosystem rather than to bacteria in isolation (60–66, **Figure 3**).

**Figure 3:**
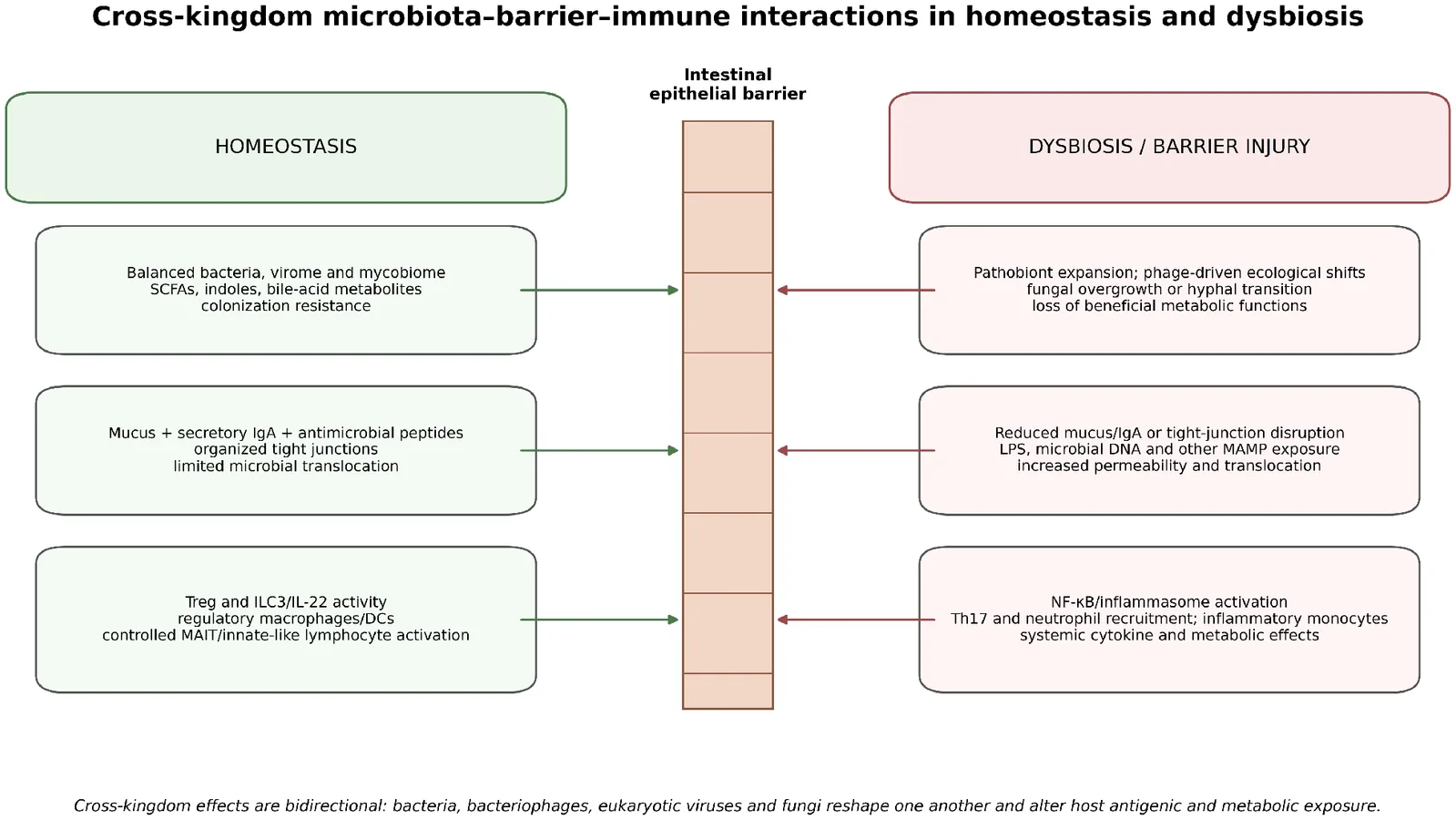
Original schematic integrating bacterial, viral and fungal ecology with epithelial barrier function and immune activation during homeostasis and dysbiosis (48–68).

## 9 Dysbiosis in immune–mediated diseases

Dysbiosis can be defined as a qualitative or quantitative alteration in microbial composition, function or localization associated with impaired host homeostasis. Common features include loss of metabolic functions, expansion of pathobionts, altered bile–acid or tryptophan metabolism, abnormal IgA coating and impaired barrier integrity. These features may shift mucosal immunity from tolerance toward inflammation, but they are neither universal nor necessarily causal across diseases (67, 68).

The gut microbiota produces neurotransmitters such as serotonin and GABA, which can influence the gut–brain axis. Serotonin binds to receptors on intestinal nerve cells and affects, among other things, bowel movements, secretion, and sensation in the gastrointestinal tract. This is just one example of how disruptions in the microbiota can cause dysregulation of the gut–brain axis and be associated with the development of diseases. In allergic and atopic disease, early–life dysbiosis may alter exposure to microbial signals required for tolerance to food and environmental antigens. Reviews by Di Costanzo et al. and Mareș et al. reach broadly concordant conclusions: both describe associations between early microbial disruption, reduced regulatory signaling and food allergy, while emphasizing that interventional evidence for microbiota modulation remains heterogeneous and insufficient for universal clinical recommendations (69, 70).

Primary studies add mechanistic detail regarding how quantitative and qualitative alterations in mucosal IgA converge to impair tolerance. Kukkonen et al. reported that higher total fecal IgA at six months was associated with a lower risk of IgE–associated allergic disease, reflecting the protective role of broad IgA–mediated immune exclusion of dietary and environmental antigens. Dzidic et al. complemented this by demonstrating that qualitative shifts in IgA target specificity – specifically altered patterns of IgA coating of the infant gut microbiota – precede the onset of allergy and asthma. These two findings directly correlate by illustrating that mucosal tolerance relies on a dual–component mechanism: quantitative IgA sufficiency must align with targeted, qualitative IgA–commensal binding, which delivers the specific signals needed for naive T–cell differentiation into FoxP3+ regulatory T cells (34, 35).

A 2026 comparison of traditional farming and urban infant cohorts further identified distinct taxon–specific IgA–coating patterns and stronger maternal milk IgA responses to selected commensals in the low–allergy farming population (106). Collectively, these studies support an association between early microbial recognition and mucosal tolerance but do not show that increasing total IgA or administering a single probiotic will prevent food allergy in all infants.

In autoimmune diseases, microbiota alterations have been reported in systemic lupus erythematosus, rheumatoid arthritis and other immune–mediated disorders. Proposed mechanisms include molecular mimicry, altered Treg/Th17 balance, increased permeability, expansion of pro–inflammatory taxa and changes in microbial metabolites. In SLE, dysbiosis has been linked to IL–6, IL–17 and interferon–related pathways, whereas in rheumatoid arthritis gut and oral microbial communities may contribute to antigenic stimulation and synovial inflammation. Disease activity and immunosuppressive therapy can themselves alter these profiles, complicating causal inference (67, 71–73).

Inflammatory bowel disease provides one of the clearest examples of a microbiota–immune–barrier interaction. Patients frequently show loss of SCFA–producing functions, pathobiont expansion, altered mucosal immunity and impaired epithelial barrier function. These findings align with the known actions of butyrate on epithelial metabolism and regulatory immunity, yet IBD cannot be reduced to dysbiosis alone because host genetics, epithelial biology, immune regulation, diet and medication all contribute to the disease state (74, 75).

Inborn errors of immunity demonstrate the bidirectionality of microbiota–immune interactions. Defects in antibody production, phagocyte function, immune regulation or lymphocyte signaling can alter microbial composition, while dysbiosis can amplify intestinal inflammation and barrier injury. Microbiota–targeted treatment in IEI therefore remains a research strategy that requires careful disease–specific safety assessment rather than routine broad application (76, 77).

Cancer immunotherapy is one of the most active translational areas. Specific gut microbial profiles have been associated with improved responses to immune checkpoint inhibitors, and antibiotic exposure near treatment initiation has frequently been associated with poorer outcomes. Mechanistic studies indicate that microbiota composition can alter antigen presentation, dendritic–cell activation, T–cell trafficking and the tumor cytokine environment. In experimental transfer models, fecal material from responding patients can improve PD–1 blockade efficacy in mice, supporting causality for at least some microbial effects, however, the taxa associated with response differ between cohorts and are not yet sufficiently reproducible for routine patient selection (78–80).

## 10 Gut–organ axes and systemic immune effects

The gut–lung, gut–skin and gut–brain axes are useful models for describing systemic microbial immunomodulation. These networks involve microbial metabolites, cytokines, immune–cell trafficking, endocrine signals and neural communication and should be understood as bidirectional systems rather than direct linear pathways (81, 82).

The gut–lung axis is relevant to asthma and respiratory infection. SCFAs can influence Treg activity, bone–marrow myelopoiesis, type I interferon responses and airway inflammation. Reduced microbial fermentation and lower SCFA availability have been linked in experimental and observational studies to impaired antiviral responses or allergic airway inflammation. Nevertheless, clinical effects of dietary and probiotic interventions remain dependent on patient age, microbial baseline, strain selection and disease phenotype (83–85).

The gut–skin axis is implicated in atopic dermatitis, psoriasis, chronic urticaria and other inflammatory skin disorders. Proposed mechanisms include intestinal permeability, circulating inflammatory mediators, altered SCFA, bile–acid and tryptophan metabolism, and systemic Th2 or Th17 polarization. These pathways are biologically coherent but differ between diseases, and clinical trials have not established a universal microbiota–directed dermatological therapy (86–88).

The gut–brain axis integrates the enteric and central nervous systems with endocrine, immune and microbial pathways. Microorganisms can influence neuroactive signaling by modifying tryptophan availability, SCFA concentrations, vagal activity and the production or metabolism of serotonin, dopamine, GABA and glutamate. Most peripheral serotonin is generated in the intestine by enterochromaffin cells, and microbial signals can alter this synthesis, however, peripheral serotonin does not freely cross the blood–brain barrier, so its central effects are indirect. GABA–producing pathways have been described in several *Lactobacillus*, *Bifidobacterium* and *Bacteroides* species, while *Enterococcus* and *Escherichia* species can influence aromatic amino–acid and catecholamine metabolism. These microbial activities may alter enteric signaling, immune–cell function, precursor availability and vagal pathways rather than simply deliver neurotransmitters directly to the brain (81, 82, 89, 90, 110).

Disease associations illustrate both the potential and the uncertainty of this axis. Dysbiosis and altered microbial metabolite profiles have been reported in depression, anxiety, neurodevelopmental disorders and Parkinson’s disease, while preclinical transfer studies support causal effects for selected phenotypes. In humans, medication, diet, intestinal motility and disease–associated behavioral changes are major confounders. Therefore, bacterial populations should be interpreted as components of a metabolic and neuroimmune network rather than as disease–specific microbial signatures (81, 82, 89, 90, 110).

## 11 Translational and clinical implications

Probiotics, prebiotics, synbiotics, postbiotics, diet and fecal microbiota transplantation (FMT) attempt to modify microbial ecology or microbial–derived signaling, but they are not interchangeable interventions. Probiotics are defined by strain–level properties and viable dose, prebiotics rely on selective utilization by the resident microbiota, synbiotics require a biologically rational pairing of organism and substrate, and postbiotics contain inanimate microorganisms or their components and therefore may offer manufacturing and safety advantages but still require defined composition and mechanism (91, 92).

For probiotics, mechanistic studies support pathogen exclusion, epithelial–barrier support, metabolite production, dendritic–cell modulation and regulatory T–cell responses. Clinical findings, however, vary markedly by strain, disease, dose, formulation and endpoint. This heterogeneity explains why positive results with one organism or mixture cannot be generalized to the probiotic category as a whole. The same limitation applies to allergy prevention and immune–mediated diseases, where some studies report benefit while others do not reproduce clinically meaningful effects (69, 70, 91, 92).

Prebiotic and dietary interventions illustrate the importance of the baseline ecosystem. Fibers can increase substrate availability for SCFA production, but the magnitude of the metabolic response depends on whether the recipient microbiota contains the required degradative pathways. In the randomized dietary study by Wastyk et al., a fermented–food diet increased microbial diversity and was accompanied by reduced inflammatory markers, whereas the high–fiber intervention produced heterogeneous immune responses related in part to baseline microbiota characteristics. These findings are not contradictory, rather, they demonstrate that different microbiota–directed dietary strategies engage distinct ecological and immune mechanisms (93, 94).

Postbiotics are attractive because they may reduce risks associated with administering viable organisms and can potentially be standardized around defined microbial structures, metabolites or inactivated preparations. Nevertheless, the term encompasses heterogeneous products and clinical evidence remains less mature than the mechanistic literature. Future trials should therefore specify the active preparation, manufacturing process, stability, immune target and indication rather than extrapolating effects from probiotics (91, 92).

FMT has the clearest established clinical role in recurrent *Clostridioides difficile* infection. In this setting, restoration of ecological diversity and colonization resistance can produce substantial clinical benefit. Outside recurrent *C. difficile* infection, including ulcerative colitis, metabolic disease and cancer immunotherapy, FMT remains investigational or limited to specialized settings. Donor selection, infectious and non–infectious safety, long–term monitoring, product standardization and mechanistic biomarkers are central unresolved issues (95, 96).

Taken together, the intervention literature contains both aligned and conflicting results because the term ‘microbiota modulation’ covers biologically different strategies and because host baseline microbiota, diet, medication and immune status modify treatment response. The most important gap is therefore not the absence of candidate interventions but the lack of standardized, indication–specific trials that connect an intervention to a defined microbial change, an immune mechanism and a clinically meaningful endpoint. Multi–omics and immune profiling may eventually enable patient stratification, but composite signatures will require external validation before routine use (97–99).

Research demonstrates the diverse bioactivity of biotics, manifested by microbiome modulation and the restoration of intestinal barrier integrity. Nevertheless, further analyses are required to comprehensively understand the signaling pathways underlying their mechanisms of action. Furthermore, the heterogeneity of existing studies – which involve diverse microbial strains, target populations, intervention protocols, and methodologies – demands a precise evaluation of their impact on long–term immunological outcomes, optimal intervention timing, and strain–specific efficacy. The capacity to restore a healthy microbiota and support immune tolerance mechanisms varies in effectiveness depending on the timing of therapy initiation, the formulations used, and the specific strains involved (92–94).

In the context of fecal microbiota transplantation (FMT), future research must target the detailed definition of functional profiles, alongside the evaluation of safety and efficacy across diverse disease entities. Current FMT protocols are highly heterogeneous, which hinders direct comparison of outcomes. Issues such as minimizing risks for recipients, the durability of clinical response, and optimal dosing regimens remain to be clarified. Additionally, there is still a lack of clear criteria (biomarkers) to predict which patients will derive the greatest benefit from this therapy (94, 95).

## 12 Limitations and future directions

The principal limitation of microbiome research remains the difficulty of separating correlation from causation. Microbial changes observed in disease may be causal drivers, consequences of inflammation or therapy, or markers of diet and lifestyle. Cross–sectional associations should therefore not be translated directly into therapeutic claims without mechanistic and interventional validation (100, 101).

Research heterogeneity is substantial. Diet, geography, age, sex, medication exposure, comorbidities, sample type, storage conditions, sequencing platform, bioinformatic pipeline and statistical model can all alter the observed microbiome signal. Lack of standardization limits reproducibility and biomarker development (102, 103).

The present article is a narrative review and is itself subject to limitations inherent to this design.

The search was structured but not exhaustive, no formal systematic risk–of–bias tool was applied, and study inclusion was guided by mechanistic and clinical relevance. The synthesis should therefore be interpreted as an integrative framework rather than a quantitative estimate of effect size.

Future research should prioritize longitudinal cohorts, controlled microbiota–modulating interventions, strain–level and functional resolution, metabolite measurement, immune phenotyping and causal modeling. Multi–omics integration is particularly important because microbial taxonomy alone often does not explain immune function. Combining metagenomics with metabolomics, transcriptomics, proteomics and immune–cell profiling may identify reproducible microbiota–immune modules (102–105).

Clinically, promising applications include prediction of immunotherapy response, flare prediction in inflammatory bowel disease, risk stratification in immune–mediated disorders and monitoring of dietary or microbial interventions. To reach routine practice, microbiome biomarkers must be reproducible, interpretable, cost–effective and demonstrably superior to existing clinical tools (97–105).

## 13 Conclusion

The microbiota is an integral regulator of immune homeostasis. It influences immunity through PRR signaling, microbial metabolites, epithelial barrier regulation, IgA responses, cross–kingdom ecological interactions and effects on innate, adaptive and innate–like lymphocytes. A balanced microbial ecosystem supports tolerance and barrier competence, whereas dysbiosis can amplify allergic, autoimmune, inflammatory, infectious and neoplastic processes. The major microbiota–immune mechanisms and their clinical relevance are summarized in **Table 3**.

**Table 3:** Clinically relevant microbiota–immune mechanisms.

Mechanism	Main immune effect	Clinical relevance
SCFAs	Treg induction, epithelial support, macrophage and dendritic–cell modulation	IBD, allergy, asthma, immunometabolism
Tryptophan–AhR pathway	IL–22 production, barrier protection, Treg/Th17 and ILC3 regulation	Mucosal inflammation, infection susceptibility
Secondary bile acids	FXR/TGR5/PXR/VDR signaling, macrophage and Th17/Treg modulation	IBD, metabolic inflammation, liver–gut immunity
Secretory IgA	Antigen exclusion, microbiota containment and mucosal tolerance	Food allergy, early–life immune development
MR1–MAIT pathway	Recognition of microbial vitamin–pathway metabolites	Mucosal immune maturation, infection and inflammatory signaling
Barrier integrity	Restriction of microbial translocation and systemic MAMP exposure	Cirrhosis, IBD, sepsis risk, systemic inflammation
Cross–kingdom interactions	Bacteria–virus–fungi modulation of antigenic and metabolic signals	IBD, fungal inflammation, post–antibiotic states
Microbiota–gut–brain signaling	Neuroactive metabolite, immune, endocrine and vagal pathways	Neuroinflammation and neuropsychiatric disease research

Microbiota–based interventions should be presented with appropriate caution. Probiotics, prebiotics, synbiotics, postbiotics, diet and FMT are heterogeneous strategies whose efficacy depends on indication, product composition, baseline microbial ecology, host immune status and clinical endpoint.

The microbiome is therefore best described as a promising therapeutic and biomarker platform rather than a universally established treatment target.

Future progress will depend on moving from taxonomic associations toward experimentally validated functions and clinically testable pathways. Standardized sampling, longitudinal designs, mechanistic intervention studies and integration of microbial, metabolic and immune data are required to translate microbiota–immune biology into reliable personalized medicine.
